# Mitigating Concerns Over Transport Delays: An Analysis of Synovial Fluid Culture Results in Arthroplasty

**DOI:** 10.7759/cureus.39751

**Published:** 2023-05-30

**Authors:** Carl Deirmengian, Krista Toler, Van Thai-Paquette, Simmi Gulati, Alex McLaren

**Affiliations:** 1 Orthopaedic Surgery, The Rothman Orthopaedic Institute, Philadelphia, USA; 2 Orthopaedic Surgery, Thomas Jefferson University, Philadelphia, USA; 3 CD Diagnostics & CD Laboratories, Zimmer Biomet, Warsaw, USA; 4 Orthopaedic Surgery, University of Arizona College of Medicine - Phoenix, Phoenix, USA

**Keywords:** clinical laboratory, culture technique, periprosthetic joint infection, knee arthroplasty, synovial fluid analysis

## Abstract

Introduction: There is a concern in the field of arthroplasty that synovial fluid transport delays may reduce the accuracy of synovial fluid culture. However, synovial fluid samples collected in the office, and sometimes in a hospital setting, often require transport to a third-party central or specialty laboratory, causing delays in the initiation of culture incubation. This study investigated the impact of transportation delays on synovial fluid culture results.

Methods: A retrospective review of prospectively collected data at one clinical laboratory, from 2016 to 2022, was conducted. A total of 125,270 synovial fluid samples from knee arthroplasties, from 2,858 different US institutions, were transported to a single clinical laboratory for diagnostic testing including synovial fluid culture (blood culture bottles). Synovial fluid to be cultured was transported in red top tubes without additives. Samples were grouped into six-time cohorts based on the number of days between aspiration and culture initiation (1-day-delay to 6-day-delay). Metrics such as culture positivity, false-positive culture rate, culture sensitivity, and proportional growth of top genera of organisms were assessed across the cohorts.

Results: Of the 125,270 samples in this study, 71.2% were received the day after aspiration (1-day-delay), with an exponential decrease in samples received on each subsequent day. Culture-positive rates for synovial fluid samples received after 1, 2, 3, 4, 5, and 6 days of transport time were 12.2%, 13.3%, 13.5%, 13.1%, 11.6%, and 11.0%, respectively. The maximum absolute difference in culture-positive rate compared to the 1-day-delay cohort was an increase of 1.3% in the 3-day-delay cohort, which was not considered a clinically meaningful difference.

The estimated false-positive culture rate remained relatively consistent across time cohorts, with values of 0.3%, 0.4%, 0.3%, 0.2%, 0.5%, and 0.5% for 1, 2, 3, 4, 5, and 6 days of transport time, respectively. None of the cohorts showed a statistically significant difference after adjustment for multiplicity compared to the 1-day-delay cohort. Culture sensitivity was estimated at 68.2%, 67.2%, 70.5%, 70.7%, 65.9%, and 70.7% for 1, 2, 3, 4, 5, and 6 days of transport time, respectively. None of the cohorts showed a statistically significant difference after adjustment for multiplicity compared to the 1-day-delay cohort. Organism proportions were consistent across time cohorts, with Staphylococcus being the most commonly identified organism. No statistically significant differences were found in the proportional contribution of major genera across the cohorts.

Conclusions: Synovial fluid culture exhibited surprisingly consistent results despite variable transport time to the destination laboratory, with differences that have minimal clinical importance. While the authors of this study advocate for short transport times as a best practice to expedite diagnosis, it appears that concerns regarding the rapid degradation of culture results due to synovial fluid transportation is unwarranted.

## Introduction

Synovial fluid culture serves as a crucial diagnostic test in detecting native septic arthritis and periprosthetic joint infection (PJI). Although recent diagnostic trends incorporate numerous biomarkers to identify culture-negative infections, a positive culture result remains the most persuasive evidence of an infection in the joint. Major PJI definitions, such as those proposed by the Musculoskeletal Infection Society (MSIS) [1], International Consensus Meeting [2], European Bone and Joint Infection Society (EBJIS) [3], and Infectious Disease Society of America (IDSA) [4], include the presence of two positive cultures of the same organism as a major criterion for diagnosing infection.

Several factors may contribute to the failure of synovial fluid culture to yield an organism from an infected joint. For example, microorganisms may form biofilms [5], which not only reduce the synovial fluid count of the microorganism but also potentially alter the organism's replication characteristics, making laboratory culture challenging. Furthermore, natural antimicrobial molecules [6] present in synovial fluid may inhibit microorganism growth in the culture. Lastly, it is speculated that poor sample collection techniques, such as low volumes, suboptimal temperatures, or delays in culture initiation, might decrease culture positivity yields.

In contemporary healthcare, synovial fluid samples are often transported to microbiology laboratories for definitive testing. In some instances, the laboratory is located in the same building as the synovial fluid aspirate, resulting in minimal delays in initiating cultures. However, in other cases, the microbiology laboratory may be situated in a different building or institution, necessitating the involvement of courier or national mail services. Moreover, insurance carriers covering the cost of synovial fluid aspirate analysis may require the sample to be sent to a specific off-site laboratory to qualify for reimbursement. It remains uncertain whether short delays in culture initiation significantly impact the successful culture of organisms from synovial fluid.

This study investigated the impact of transportation delays on synovial fluid culture results.

## Materials and methods

This study conducted a retrospective analysis on prospectively collected data from a single clinical microbiology laboratory specializing in synovial fluid analysis (Zimmer Biomet, CD Laboratories, Towson, MD). The data used for this investigation were compiled prospectively from 2016 to 2022. This study has never been submitted for publication to any other journal. An abstract for this study was presented at the meeting of the Musculoskeletal Infection Society (Aug 6, 2022, Pittsburgh, PA) and the meeting of the American Association of Orthopaedic Surgeons (Mar 7, 2023, Las Vegas, NV).

Data collection methods

Data collected for this study were digitally transferred directly from clinical diagnostic instrumentation into a deidentified research database, approved by the Institutional Review Board (WCG IRB, Puyallup, WA). The digital transmission of results from laboratory devices to a lab information system, CGM LabDAQ, is designed to ensure optimal data integrity. This system is built on Microsoft SQL Server, a relational database management system (RDBMS) that is ACID-compliant and adheres to industry standards. Database searches are conducted to isolate specific subsets of data. Additionally, any protected health information (PHI) is deidentified as needed by excluding it from the search queries. If it is impossible to exclude such information during the query, then a manual process of deletion is implemented prior to the data being shared. with rigorous verification and error-checking procedures in place. The resulting database of prospectively compiled data was utilized for retrospective analysis.

Synovial fluid samples and testing

The clinical laboratory received knee arthroplasty samples from 2,858 institutions for the purpose of clinical diagnostic testing in the context of patient care. Samples were collected in laboratory tubes specific to the test ordered and packed at room temperature in a transport kit supplied by the central laboratory. The collection kit was then mailed via a national overnight service at ambient temperature to the central laboratory for testing. Synovial fluid to be cultured was transported in red top tubes without additives.

**Figure 1:**
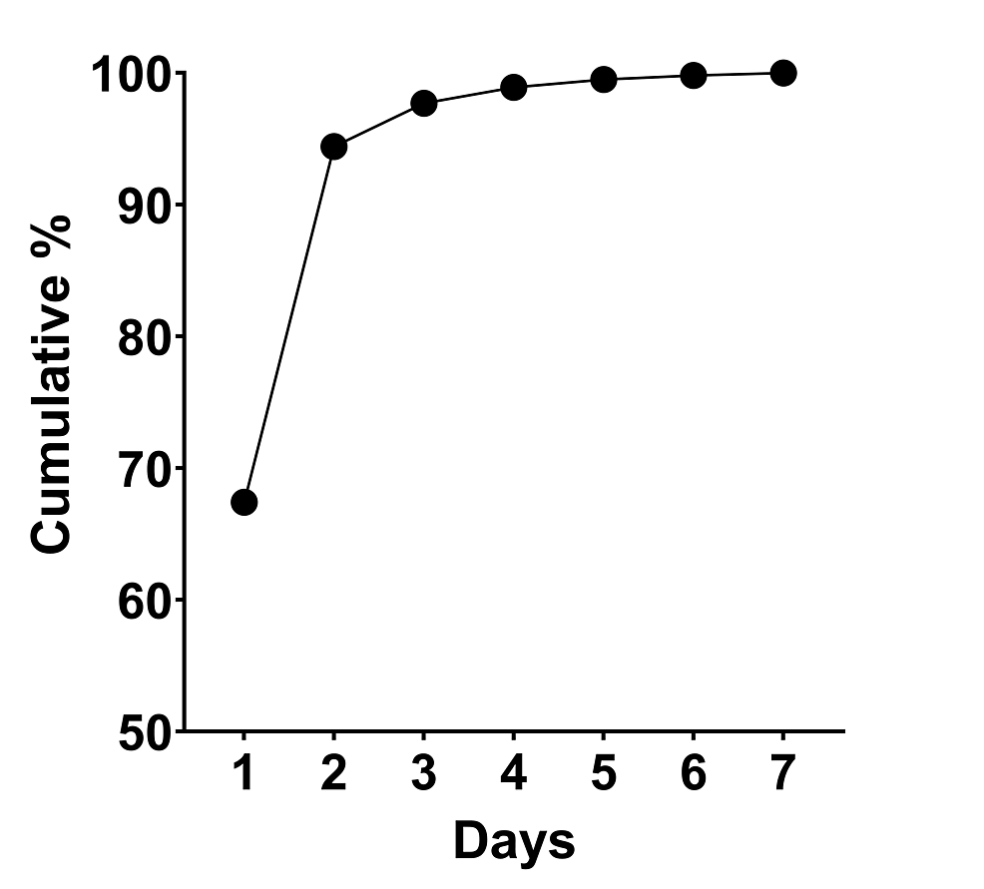
Cumulative culture positivity versus days after culture initiation. For all positive cultures in this study, the time to organism growth was recorded. The cumulative percent of culture-positive samples yielding growth was plotted against each day after culture initiation. By day 2 after culture initiation, 94.4% of all culture-positive samples had already yielded growth.

All sample tests were conducted on the day of receipt at a single Clinical Laboratory Improvements Amendments (CLIA) certified laboratory, authorized to perform testing in general immunology, hematology, routine chemistry, and bacteriology. Synovial fluid cultures were processed using BacT/Alert (Biomerieux, Inc; https://www.biomerieux-usa.com) facultative aerobic and anerobic bottles, with the BacT/Alert system monitoring growth presence. Samples were incubated for up to 7 days, with 94.4% of all positive cultures in this study demonstrating growth by day 2, and 99.5% of all positive cultures in this study demonstrating growth by day 5 after culture initiation (Figure 1).

Laboratory data collection

In addition to individual laboratory test results, additional data were prospectively collected to enable the analysis of time-sensitive metrics. The dates of sample aspiration by the institution and sample receipt by the laboratory were recorded, facilitating the calculation of transport time (days) from aspiration to laboratory testing. Each sample was also annotated by institution and source of aspiration (joint) with laterality.

Inclusion criteria included a date of aspirate from 2016 to 2022, knee arthroplasty source, culture results availability, alpha-defensin results availability, quality metrics met [7] (including red blood cells < 1M cells/µL and absorbance at 280 nm between 0.342 and 1.19), and synovial fluid transport time ranging from 1 to 6 days. A total of 125,270 samples from the laboratory database met the inclusion criteria and were evaluated for this study.

Data analysis

All samples were categorized into different time cohorts based on the number of days elapsed between sample collection and synovial fluid culture initiation, including transport time to the laboratory. For instance, the 1-day-delay time cohort included all synovial fluid samples received at the laboratory within 24 h of aspiration. This categorization resulted in six-time cohorts, representing sample cohorts with delays from 1 to 6 days between aspiration and culture initiation.

All samples were also assigned a 2018 ICM point score. The 2018 ICM definition of PJI was modified by replacing the serum C-reactive protein (CRP) (two points; cutoff = 10 mg/L) with the synovial fluid CRP (2 points; cutoff = 4.45 mg/L). Several previous studies have demonstrated the equivalence or superiority of synovial fluid CRP compared to serum CRP in the diagnosis of PJI [8-9]. A complete set of synovial fluid laboratory data (CRP, alpha-defensin, synovial fluid white blood cell count, synovial fluid polymorphonuclear cell percent, and culture) for ICM scoring was available for 99.3% (124,393/125,270) of samples. Samples were classified as Not Infected (75.3%, N=94,296), Inconclusive (7.1%, N=8,894), or Infected (17.6%, N=22,080). Clinical and laboratory data which was not part of the synovial fluid results were unavailable and not included in ICM scoring. 

Various evaluation metrics were used to identify changes in culture results associated with the delay of initiating cultures across 1-day-delay to 6-day-delay cohorts. First, the overall sample culture-positive rate was assessed across all cohorts to identify changes in culture positivity associated with increasing delays to culture initiation. Second, the false-positive culture rate across time cohorts was estimated by calculating the proportion of culture-positive samples among ICM-Not Infected samples. Third, the sensitivity of culture across time cohorts was estimated by calculating the proportion of culture-positive samples among ICM-Infected samples. Finally, the proportional growth of top genera of organisms was assessed across time cohorts, to identify potential organism growth perturbations associated with delay of culture initiation. 

When assessing for clinical differences, any changes in the absolute culture-positivity percentage greater than 3% were considered meaningful. The chi-squared test with Yates’ correction was utilized to determine if differences between two proportions reached statistical significance, with correction for multiplicity when necessary.

## Results

Cohort characteristics

**Figure 2:**
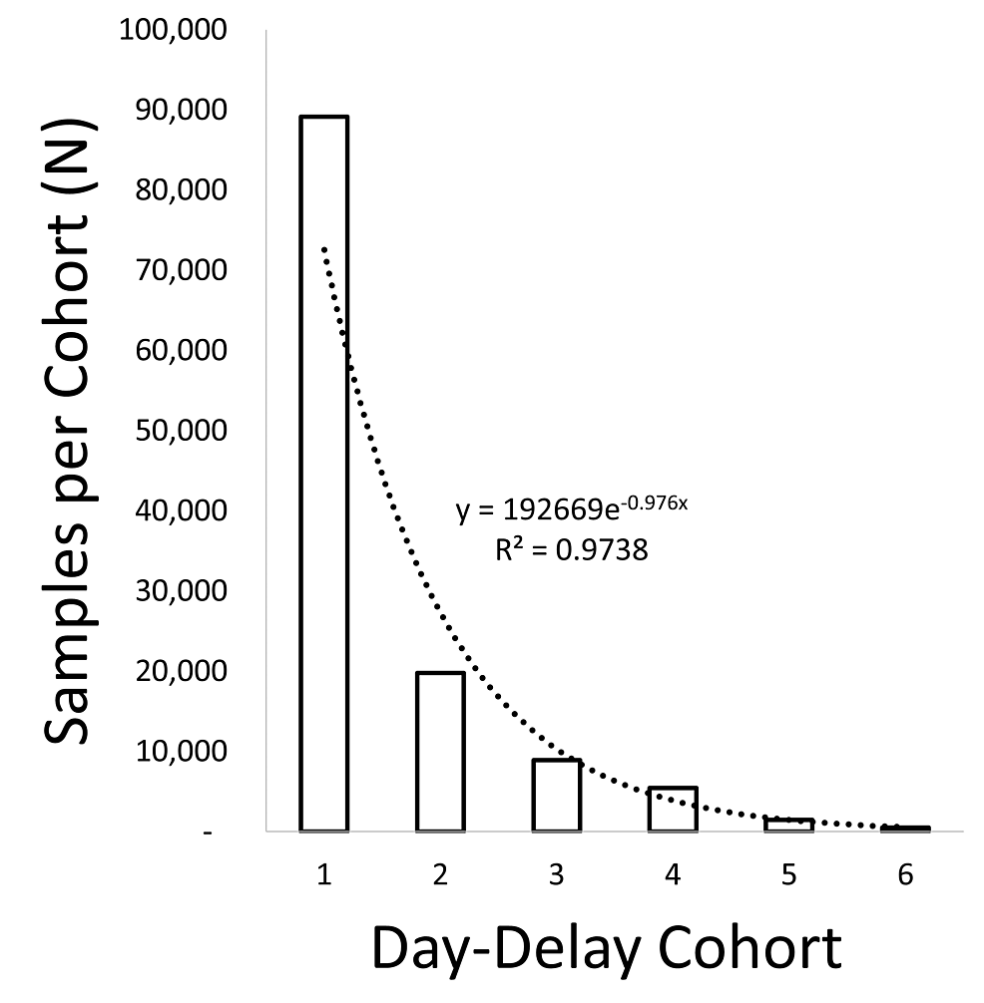
Number (N) of samples per study cohort. The number of samples (N) in each cohort is graphed, from 1-day-delay to 6-day-delay. There is an exponential decrease in samples per cohort, as most samples are received within 24 h of aspiration.

From the entire sample set of 125,270 synovial fluid samples included in this study, the transport time from joint aspiration to synovial fluid sample testing had a mean of 1.5 days and a median of 1 day. Samples with a transport time of one day (1-day-delay cohort) accounted for 71.2% (N=89,134) of all samples. An exponential decline (R2=0.97) in the number of samples received on each additional day of sample transport was observed, with a low of 0.4% (N=501) of samples being received on day 6 (6-day-delay cohort) after aspiration (Figure 2).

Minimal variation in culture-positivity across time cohorts 

**Figure 3:**
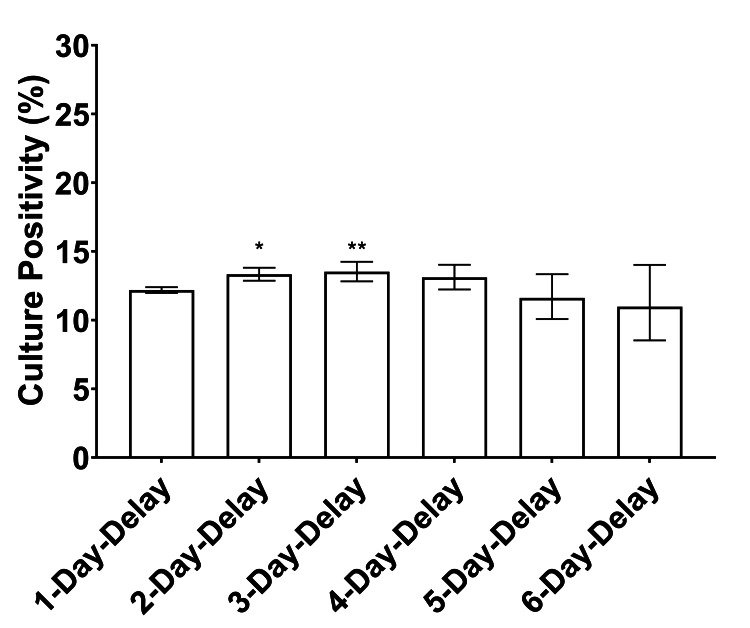
The culture-positive rate from synovial fluid varies minimally due to sample transport delay. The culture-positive rate is graphed for each cohort in the study. The (*) denotes a statistically significant increase in culture yields with p < 0.0001. The (**) denotes a statistically significant increase in culture yields with p = 0.002. Neither of these increases was deemed clinically meaningful.

The culture-positive rates of synovial fluid samples received after 1, 2, 3, 4, 5, and 6 days of transport time were 12.2%, 13.3%, 13.5%, 13.1%, 11.6%, and 11.0%, respectively. The maximum absolute divergence from the 1-day-delay culture positivity of 12.2% was an increase of 1.3% in the 3-day-delay cohort. Only the 2-day- and 3-day-delay cohorts demonstrated a statistically significant difference after adjustment for multiplicity (p < 0.001 and p = 0.002 respectively). Although comparison of the increased proportions in cohorts 2-day-delay (13.3%) and 3-day-delay (13.5%) to the proportion of 1-day-delay (12.2%) cohort yielded statistically significant differences, none of the absolute differences were large enough to be considered a clinically meaningful difference (Figure 3).

Minimal variation in estimated false-positive culture rate across time cohorts

**Figure 4:**
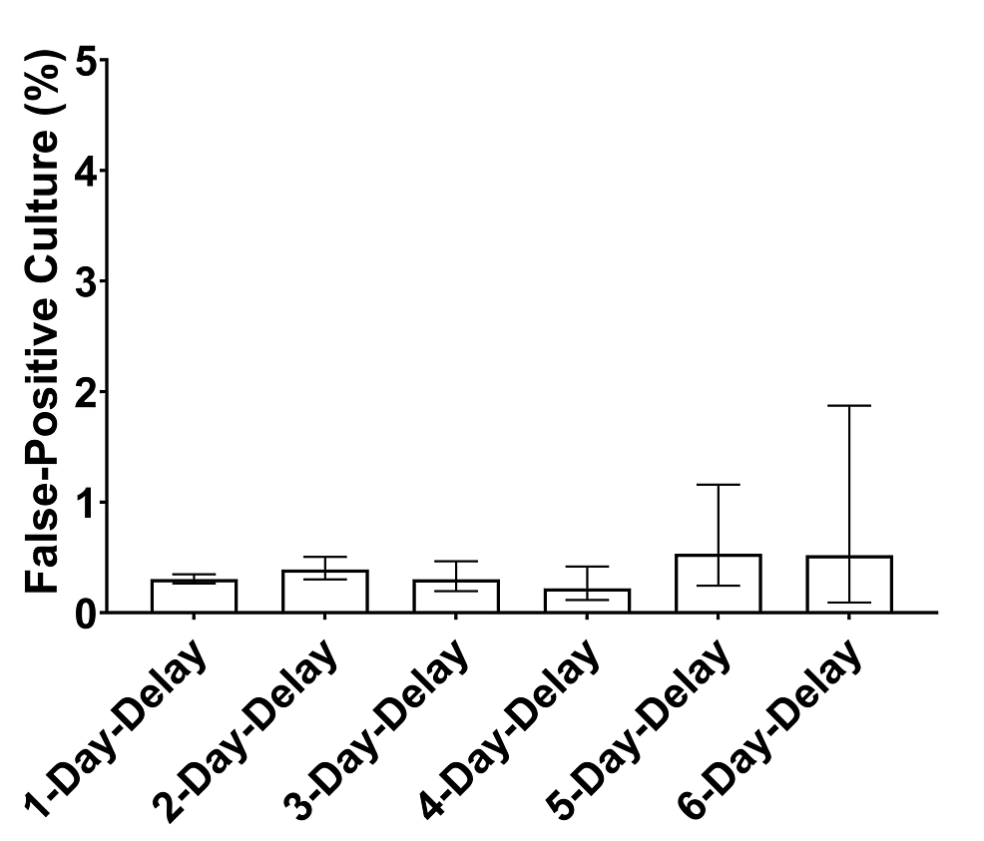
The culture false-positive rate from synovial fluid varies minimally due to sample transport delay. The culture false-positive rate is graphed, revealing no significant differences between any cohort compared to 1-day-delay.

The false-positive culture rate among synovial fluid samples received after 1, 2, 3, 4, 5, and 6 days of transport time were 0.3%, 0.4%, 0.3%, 0.2%, 0.5%, and 0.5%, respectively. When compared to the false-positive rate of 0.3% in the 1-Day-Delay cohort, none of the cohorts from 2-day-delay to 6-day-delay exhibited a statistically significant difference after adjustment for multiplicity (Figure 4).

Minimal variation in culture sensitivity across time cohorts

**Figure 5:**
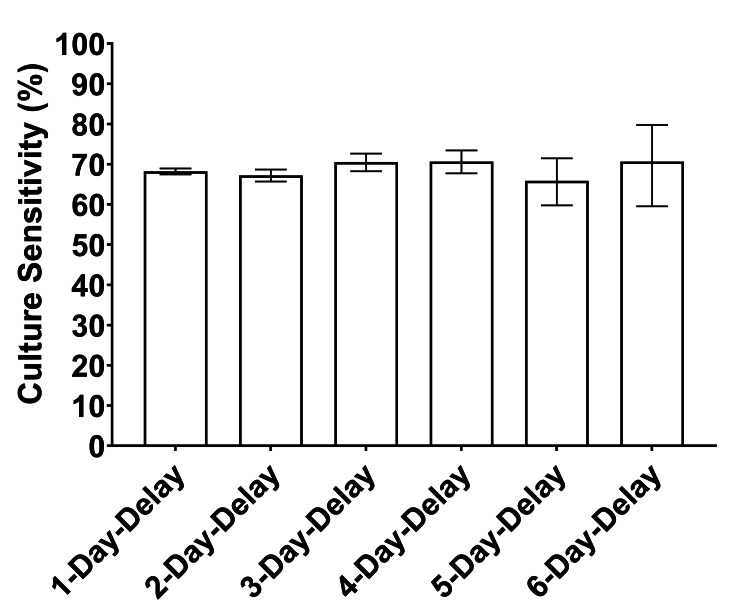
The culture sensitivity from synovial fluid varies minimally due to sample transport delay. The culture sensitivity is graphed for each cohort, revealing no significant differences between any cohort compared to 1-day-delay.

The culture sensitivity among synovial fluid samples received after 1, 2, 3, 4, 5, and 6 days of transport time were 68.2%, 67.2%, 70.5%, 70.7%, 65.9%, and 70.7%, respectively. When compared to the sensitivity of 68.3% in the 1-day-delay cohort, none of the cohorts from 2-day-delay to 6-day-delay exhibited a statistically significant difference after adjustment for multiplicity (Figure 5).

Minimal variation in organism proportions across time cohorts

There were 15,649 culture-positive samples in this study. Staphylococcus was the most commonly identified organism from the culture in this study (Table 1).

**Table 1:** Number (N) and percentage (%) of microorganism genera isolated from synovial fluid.

Genera	N (%)
Staphylococcus	9751 (61.3%)
Streptococcus	1871 (11.8%)
Enterococcus	778 (4.9%)
Candida	600 (3.8%)
Pseudomonas	565 (3.6%)
Corynebacterium	418 (2.6%)
Escherichia	377 (2.4%)

**Figure 6:**
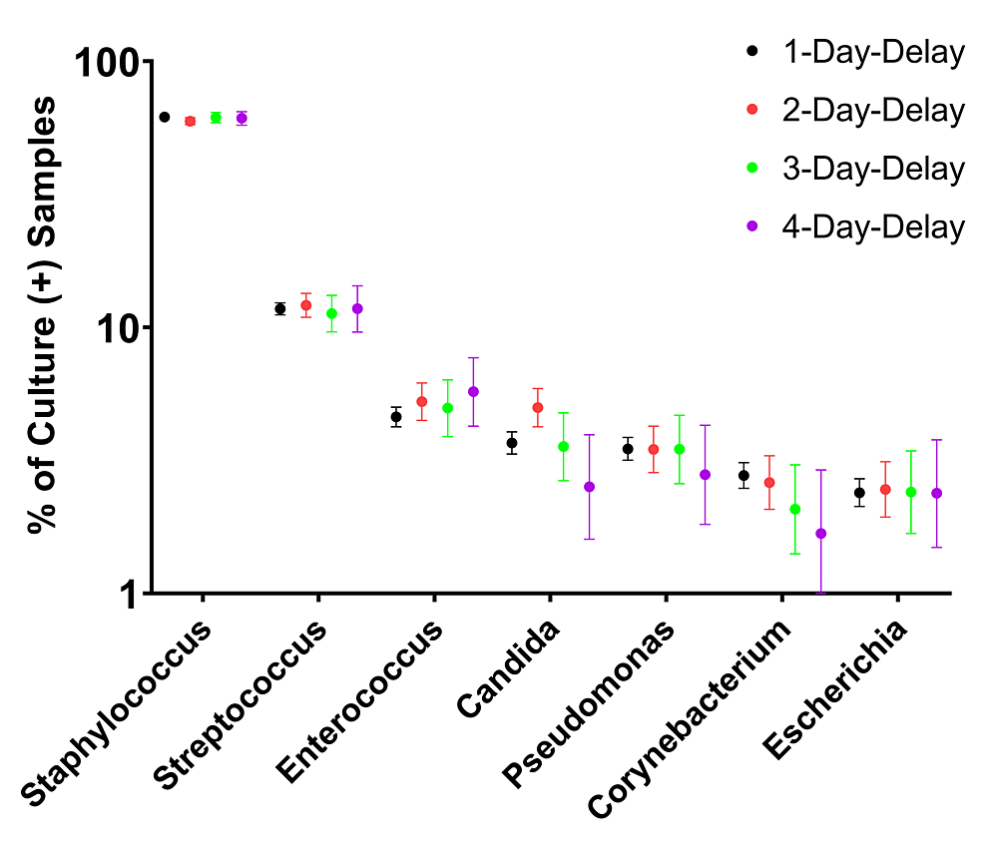
The top genera of microorganisms cultured from synovial fluid over study cohorts. The number of cultures yielding growth of each top genera was calculated as a percentage of all culture-positive samples in the study, for each cohort. The proportion of top genera growth did not vary significantly as a function of increasing delays in initiating synovial fluid culture.

There were sufficient culture-positive samples in the first four time-delay cohorts to assess organism proportions. Analysis of the proportional contribution of these major genera to each time-delay cohort did not demonstrate statistically significant differences (Figure 6).

## Discussion

This study aimed to investigate the impact of delays in initiating synovial fluid cultures by evaluating several culture-result metrics across six time-delay cohorts, ranging from a 1-day- to a 6-day-delay. This analysis holds particular relevance in light of the current practice of transporting aspirate samples to external laboratories, a requirement often imposed by insurance carriers. Our study utilized a large, high-integrity dataset from a single laboratory to assess the relationship between culture initiation delays and synovial fluid culture results. Surprisingly, we found that delays in initiating synovial fluid cultures on the order of days led to minimal changes in the results. It is important to note, however, that the majority of samples received by the laboratory arrived within one to two days of collection, with only a minority of samples requiring additional days for transportation. The authors of this study generally support the recommendation to minimize the delay in culture initiation. However, when this is not feasible or unexpected transportation delays occur, it appears that synovial fluid can still provide reliable and accurate results.

Theoretical concerns underpin the standard orthopedic teaching emphasizing the urgency of processing synovial fluid in a microbiology laboratory. Firstly, it may be reasonable to hypothesize that fluid held outside the body could compromise the viability of bacteria in the fluid, leading to decreased culture yield and sensitivity. Secondly, delays in processing synovial fluid samples could potentially result in bacterial overgrowth due to contamination, increasing the false-positive rate of synovial fluid culture. Authoritative bodies [10] and authors [11] have recommended expeditious processing of synovial fluid, suggesting that failure to do so may lead to lower culture accuracy. However, to our knowledge, no studies have examined the current standard practice of transporting synovial fluid to another institution for culture processing, nor have there been large studies attempting to understand the rate at which fluid sample degradation occurs.

The first metric employed in this study to evaluate culture results across time cohorts was culture positivity among all samples in each cohort. We demonstrated that overall culture positivity from all synovial fluid samples received by a clinical laboratory does not vary substantially as a function of days in transport. Different time-delay cohorts of synovial fluid samples, with transportation times varying from one day to six days after aspiration, exhibited culture-positivity within a relatively tight band of approximately 12%-14%. Due to the large number of samples in this study, statistically significant differences in culture yields were identified in the 2-day-delay and 3-day-delay cohorts; however, the differences did not meet the criterion of demonstrating an absolute difference of 3% to reach the threshold of a clinically meaningful difference. This result alleviates concerns that microorganisms lose their ability to grow in culture with delays in initiation, as culture positivity did not decrease with increased delays in culture initiation over six days in this study.

The second metric used in this study to evaluate culture results across time cohorts was the false-positive rate. A false-positive culture rate of 0.3% was observed among samples in the 1-day-delay cohort. Interestingly, the false-positive rate for all subsequent time cohorts, which represented delays of 2-6 days before culture initiation, remained within the range of 0.2%-0.5%. This rate exhibited no statistically significant change compared to samples in the 1-day-delay cohort. Therefore, concerns regarding contaminant overgrowth in sample tubes due to delays in initiating synovial fluid cultures are not justified.

The third metric used in this study to evaluate culture results across time cohorts was sensitivity. A culture sensitivity of 61.8% was observed among samples in the 1-day-delay cohort. Cohorts from 1-day-delay to 6-day-delay displayed a narrow range of sensitivity between 65.9% and 70.7%, with no significant differences. When using the 2018 ICM scoring system to assess the diagnosis of a sample, the sensitivity of culture does not demonstrate a decline with delays in the initiation of culture. Therefore, concerns that microorganisms from infected samples can lose their ability to grow in culture, due to a delay in the initiation of cultures, are unwarranted.

The final metric used in this study to evaluate culture results was the proportion of top microorganism genera across time cohorts. We analyzed these data considering the possibility that certain microorganisms might be more adversely affected by transport delays than others. Due to decreasing numbers in each subsequent cohort, the analysis was limited to evaluating organism proportions up to the 4-day-delay cohort. The proportion of the top genera yielded from synovial fluids, including Staphylococcus, Streptococcus, Enterococcus, Candida, Pseudomonas, Corynebacterium, and Escherichia, showed no substantial differences across the first 4 day-delay cohorts in this study. While it is possible that certain rare organisms have yields impacted by delays in culture initiation, it appears that the major genera accounting for the vast majority of PJIs are not affected by up to a 4-day delay.

This study has some limitations. The most significant is that the first-time cohort involved transportation to the lab over a 24-h period, which may already reflect the degradation of culture yields. However, we believe this is unlikely for several reasons. First, the synovial fluid culture yield among likely infected samples in this study was consistent with the existing literature [12-14]. Second, the overall false-positive culture rate in this study was quite low. Therefore, it appears unlikely that the first-time cohort in this study represents a major degradation from the standard of care. A second limitation is that the results may be specific to the specimen tube used to transport samples and the microbiological culture method. The specimen tube does not have any special organism preservatives, and the bottle technique for processing cultures is relatively standard across the country. It is important to note that the study results may not apply to other techniques or tissues; they only reference synovial fluid samples collected in a tube without preservatives and cultured using a fluid bottle culture system. More specifically, the results may not apply to other methods of culturing or samples like tissue or bone that could potentially desiccate with transport delays.

## Conclusions

Our study examined the impact of delays in initiating synovial fluid cultures using various culture result metrics across six time-delay cohorts, from 1-day- to 6-day-delays. This analysis is especially relevant due to the current practice of transporting aspirate samples to outside laboratories, which may be mandated by insurance carriers. Surprisingly, synovial fluid culture displayed highly consistent positivity, false-positive rate, sensitivity, and organism profiles despite the delay in culture initiation, with differences appearing to have minimal clinical importance. Although the authors advocate for short transport times as a best practice to expedite diagnosis, it seems that concerns regarding the rapid degradation of synovial fluid culture results due to transportation are unwarranted.
